# 

**Published:** 1899-07-08

**Authors:** 


					THE HOSPITAL. ? The standard of highest purity."?Lancet, July 8th, 1899.
CADBURY'S n* U CADBURY'S
CS
COCOA
NO DRUGS OR ALKALIES USED.
ULascoiy Western Infirmary
?rrco ? cni? Iivrinn/ini
7. Vol. XXVI. [?gg%*?)
Saturday, July 8th, 1899.
'ORFOLKaud NORW/CH HGseiTAU
jtion Terms,! _
p. 242. J PRICE TWOPENCE.

				

